# Supplementary material for: Broad-spectrum infrared thermography for detection of M2 digital dermatitis lesions on hind feet of standing dairy cattle
Source: PLoS One. 2023 Jan 17;18(1):e0280098. doi: 10.1371/journal.pone.0280098 (PMC9844892; doi:10.1371/journal.pone.0280098)
Supplement: S1 Table — (DOCX) [file pone.0280098.s001.docx]

**S1 Table. Technical data for the FLiR i3 handheld infrared thermography camera.**

| Technical Metric | FLiR i3 |
| --- | --- |
| Field of View (FOV) | 12.5° x 12.5° |
| Minimum Focus Distance | 0.6m (2 ft) |
| Spatial Resolution | 3.7 mrad |
| Thermal sensitivity/NETD | <0.15°C (0.27°F) / 150 mk |
| Image frequency | 9 Hz |
| Focus | Focus free |
| Detector type | Focal place array (FPA), uncooled microbolometer |
| Spectral range | 7.5 - 13 μm |
| IR resolution | 60 x 60 pixels |
| Display | 2.8 inch color LCD |
| Image adjustment | Automatic adjust/lock image |
| Object temperature range | -20°C to 250°C (-4°F to 482°F) |
| Accuracy | ±2°C (±3.6°F) or ±2% of reading, for ambient temperature 10°C to 35°C (50°F to 95°F) |
