# Supplementary material for: Broad-spectrum infrared thermography for detection of M2 digital dermatitis lesions on hind feet of standing dairy cattle
Source: PLoS One. 2023 Jan 17;18(1):e0280098. doi: 10.1371/journal.pone.0280098 (PMC9844892; doi:10.1371/journal.pone.0280098)
Supplement: S1 File — Full results of all regression analyses performed investigating the association between maximum infrared temperature (IRTmax) of the plantar pastern region of feet from standing dairy cattle and the presence of M2 lesions of digital dermatitis. (DOCX) [file pone.0280098.s002.docx]

**S2 Regression analyses M2 lesions. Full results of all regression analyses performed investigating the association between maximum infrared temperature (IRTmax) of the plantar pastern region of feet from standing dairy cattle and the presence of M2 lesions of digital dermatitis.**

# UNIVARIABLE LOGISTIC REGRESSION ANALYSES

# UNWASHED FEET DATASET

# univariable logistic regression for M2 yes/no and IRTmax

glm(formula = m2bin ~ IRTmax, family = "binomial", data = unwashed)

Deviance Residuals:

Min 1Q Median 3Q Max

-0.7551 -0.4317 -0.3247 -0.2180 2.6727

Coefficients:

Estimate Std. Error z value Pr(>|z|)

(Intercept) -14.16399 3.12297 -4.535 5.75e-06 ***

IRTmax 0.36878 0.09749 3.783 0.000155 ***

---

Signif. codes: 0 ‘***’ 0.001 ‘**’ 0.01 ‘*’ 0.05 ‘.’ 0.1 ‘ ’ 1

(Dispersion parameter for binomial family taken to be 1)

Null deviance: 262.99 on 528 degrees of freedom

Residual deviance: 244.06 on 527 degrees of freedom

AIC: 248.06

Number of Fisher Scoring iterations: 6

Single term deletions

Model:

m2bin ~ IRTmax

Df Deviance AIC LRT Pr(>Chi)

<none> 244.06 248.06

IRTmax 1 262.99 264.99 18.927 1.358e-05 ***

---

Signif. codes: 0 ‘***’ 0.001 ‘**’ 0.01 ‘*’ 0.05 ‘.’ 0.1 ‘ ’ 1

beta 2.5 % 97.5 %

(Intercept) 0.00 0.00 0.00

IRTmax 1.45 1.21 1.77

# univariable logistic regression for M2 yes/no and IRTmax dichotomised by the median

glm(formula = m2bin ~ factor(IRTmax_median), family = "binomial", data = unwashed)

Deviance Residuals:

Min 1Q Median 3Q Max

-0.4902 -0.4902 -0.2144 -0.2144 2.7511

Coefficients:

Estimate Std. Error z value Pr(>|z|)

(Intercept) -3.7612 0.4130 -9.108 < 2e-16 ***

factor(IRTmax_median)1 1.7028 0.4562 3.733 0.00019 ***

---

Signif. codes: 0 ‘***’ 0.001 ‘**’ 0.01 ‘*’ 0.05 ‘.’ 0.1 ‘ ’ 1

(Dispersion parameter for binomial family taken to be 1)

Null deviance: 262.99 on 528 degrees of freedom

Residual deviance: 244.45 on 527 degrees of freedom

AIC: 248.45

Number of Fisher Scoring iterations: 6

Single term deletions

Model:

m2bin ~ factor(IRTmax_median)

Df Deviance AIC LRT Pr(>Chi)

<none> 244.45 248.45

factor(IRTmax_median) 1 262.99 264.99 18.538 1.666e-05 ***

---

Signif. codes: 0 ‘***’ 0.001 ‘**’ 0.01 ‘*’ 0.05 ‘.’ 0.1 ‘ ’ 1

beta 2.5 % 97.5 %

(Intercept) 0.02 0.01 0.05

factor(IRTmax_median)1 5.49 2.40 14.83

# univariable logistic regression M2 yes/no and locomotion score

glm(formula = m2bin ~ factor(locomotion score), family = "binomial", data = unwashed)

Deviance Residuals:

Min 1Q Median 3Q Max

-0.7585 -0.3954 -0.3954 -0.3954 2.2750

Coefficients:

Estimate Std. Error z value Pr(>|z|)

(Intercept) -2.5096 0.3288 -7.632 2.31e-14 ***

factor(locomotion score)2 0.6904 0.5494 1.257 0.209

factor(locomotion score)3 -16.0565 1423.3565 -0.011 0.991

factor(locomotion score)4 1.4110 0.8802 1.603 0.109

factor(locomotion score)5 -16.0565 6522.6386 -0.002 0.998

---

Signif. codes: 0 ‘***’ 0.001 ‘**’ 0.01 ‘*’ 0.05 ‘.’ 0.1 ‘ ’ 1

(Dispersion parameter for binomial family taken to be 1)

Null deviance: 122.13 on 205 degrees of freedom

Residual deviance: 114.74 on 201 degrees of freedom

(323 observations deleted due to missingness)

AIC: 124.74

Number of Fisher Scoring iterations: 17

Single term deletions

Model:

m2bin ~ factor(locomotion score)

Df Deviance AIC LRT Pr(>Chi)

<none> 114.73 124.73

factor(locomotion score) 4 122.13 124.13 7.3941 0.1165

beta 2.5 % 97.5 %

(Intercept) 0.08 0.04 1.500000e-01

factor(locomotion score)2 1.99 0.64 5.750000e+00

factor(locomotion score)3 0.00 NA 8.567738e+31

factor(locomotion score)4 4.10 0.55 2.072000e+01

factor(locomotion score)5 0.00 NA Inf

# univariable logistic regression M2 yes/no and lame (locomotion score 3+4+5)

glm(formula = m2bin ~ factor(lame), family = "binomial", data = unwashed)

Deviance Residuals:

Min 1Q Median 3Q Max

-0.4366 -0.4366 -0.4366 -0.4366 2.3272

Coefficients:

Estimate Std. Error z value Pr(>|z|)

(Intercept) -2.3026 0.2622 -8.782 <2e-16 ***

factor(lame)1 -0.3365 0.7775 -0.433 0.665

---

Signif. codes: 0 ‘***’ 0.001 ‘**’ 0.01 ‘*’ 0.05 ‘.’ 0.1 ‘ ’ 1

(Dispersion parameter for binomial family taken to be 1)

Null deviance: 122.13 on 205 degrees of freedom

Residual deviance: 121.93 on 204 degrees of freedom

(323 observations deleted due to missingness)

AIC: 125.93

Number of Fisher Scoring iterations: 5

Single term deletions

Model:

m2bin ~ factor(lame)

Df Deviance AIC LRT Pr(>Chi)

<none> 121.93 125.93

factor(lame) 1 122.13 124.13 0.20171 0.6533

beta 2.5 % 97.5 %

(Intercept) 0.10 0.06 0.16

factor(lame)1 0.71 0.11 2.70

# univariable logistic regression M2 yes/no and cleanliness score

glm(formula = m2bin ~ factor(cleanliness score), family = "binomial", data = unwashed)

Deviance Residuals:

Min 1Q Median 3Q Max

-0.5679 -0.5679 -0.2944 -0.2944 2.5140

Coefficients:

Estimate Std. Error z value Pr(>|z|)

(Intercept) -2.4849 0.7360 -3.376 0.000735 ***

factor(cleanliness score)2 -0.6318 0.8312 -0.760 0.447194

factor(cleanliness score)3 0.7419 0.7909 0.938 0.348225

factor(cleanliness score)4 -15.0812 1495.2958 -0.010 0.991953

---

Signif. codes: 0 ‘***’ 0.001 ‘**’ 0.01 ‘*’ 0.05 ‘.’ 0.1 ‘ ’ 1

(Dispersion parameter for binomial family taken to be 1)

Null deviance: 161.04 on 291 degrees of freedom

Residual deviance: 151.16 on 288 degrees of freedom

(237 observations deleted due to missingness)

AIC: 159.16

Number of Fisher Scoring iterations: 16

Single term deletions

Model:

m2bin ~ factor(cleanliness score)

Df Deviance AIC LRT Pr(>Chi)

<none> 151.16 159.16

factor(cleanliness score) 3 161.04 163.04 9.8741 0.01967 *

---

Signif. codes: 0 ‘***’ 0.001 ‘**’ 0.01 ‘*’ 0.05 ‘.’ 0.1 ‘ ’ 1

beta 2.5 % 97.5 %

(Intercept) 0.08 0.01 2.800000e-01

factor(cleanliness score)2 0.53 0.12 3.710000e+00

factor(cleanliness score)3 2.10 0.54 1.397000e+01

factor(cleanliness score)4 0.00 NA 3.861029e+30

# univariable logistic regression M2 yes/no and dried manure (cleanliness score 3+4)

glm(formula = m2bin ~ factor(dried manure), family = "binomial", data = unwashed)

Deviance Residuals:

Min 1Q Median 3Q Max

-0.5463 -0.5463 -0.3107 -0.3107 2.4719

Coefficients:

Estimate Std. Error z value Pr(>|z|)

(Intercept) -3.0068 0.3414 -8.806 < 2e-16 ***

factor(dried manure)1 1.1799 0.4467 2.642 0.00825 **

---

Signif. codes: 0 ‘***’ 0.001 ‘**’ 0.01 ‘*’ 0.05 ‘.’ 0.1 ‘ ’ 1

(Dispersion parameter for binomial family taken to be 1)

Null deviance: 161.04 on 291 degrees of freedom

Residual deviance: 153.85 on 290 degrees of freedom

(237 observations deleted due to missingness)

AIC: 157.85

Number of Fisher Scoring iterations: 5

Single term deletions

Model:

m2bin ~ factor(dried manure)

Df Deviance AIC LRT Pr(>Chi)

<none> 153.85 157.85

factor(dried manure) 1 161.04 163.04 7.1841 0.007355 **

---

Signif. codes: 0 ‘***’ 0.001 ‘**’ 0.01 ‘*’ 0.05 ‘.’ 0.1 ‘ ’ 1

beta 2.5 % 97.5 %

(Intercept) 0.05 0.02 0.09

factor(dried manure)1 3.25 1.37 8.09

# univariable logistic regression M2 yes/no and farm

glm(formula = m2bin ~ factor(farm), family = "binomial", data = unwashed)

Deviance Residuals:

Min 1Q Median 3Q Max

-0.5143 -0.4523 -0.3231 -0.2604 2.7825

Coefficients:

Estimate Std. Error z value Pr(>|z|)

(Intercept) -3.3673 0.7192 -4.682 2.84e-06 ***

factor(farm)2 1.4112 0.7738 1.824 0.0682 .

factor(farm)3 1.1388 0.8219 1.386 0.1659

factor(farm)4 -0.4829 1.2403 -0.389 0.6971

factor(farm)5 0.4406 0.7778 0.566 0.5711

---

Signif. codes: 0 ‘***’ 0.001 ‘**’ 0.01 ‘*’ 0.05 ‘.’ 0.1 ‘ ’ 1

(Dispersion parameter for binomial family taken to be 1)

Null deviance: 262.99 on 528 degrees of freedom

Residual deviance: 252.72 on 524 degrees of freedom

AIC: 262.72

Number of Fisher Scoring iterations: 6

Single term deletions

Model:

m2bin ~ factor(farm)

Df Deviance AIC LRT Pr(>Chi)

<none> 252.72 262.72

factor(farm) 4 262.99 264.99 10.269 0.03613 *

---

Signif. codes: 0 ‘***’ 0.001 ‘**’ 0.01 ‘*’ 0.05 ‘.’ 0.1 ‘ ’ 1

beta 2.5 % 97.5 %

(Intercept) 0.03 0.01 0.11

factor(farm)2 4.10 1.09 26.71

factor(farm)3 3.12 0.72 21.53

factor(farm)4 0.62 0.03 6.63

factor(farm)5 1.55 0.41 10.16

# WASHED FEET DATASET

# univariable logistic regression for M2 yes/no and IRTmax

glm(formula = m2bin ~ IRTmax, family = "binomial", data = washed)

Deviance Residuals:

Min 1Q Median 3Q Max

-0.6753 -0.4455 -0.3564 -0.2478 2.5879

Coefficients:

Estimate Std. Error z value Pr(>|z|)

(Intercept) -12.29236 2.94897 -4.168 3.07e-05 ***

IRTmax 0.30964 0.09192 3.368 0.000756 ***

---

Signif. codes: 0 ‘***’ 0.001 ‘**’ 0.01 ‘*’ 0.05 ‘.’ 0.1 ‘ ’ 1

(Dispersion parameter for binomial family taken to be 1)

Null deviance: 287.90 on 557 degrees of freedom

Residual deviance: 272.32 on 556 degrees of freedom

AIC: 276.32

Number of Fisher Scoring iterations: 6

Single term deletions

Model:

m2bin ~ IRTmax

Df Deviance AIC LRT Pr(>Chi)

<none> 272.32 276.32

IRTmax 1 287.90 289.90 15.582 7.901e-05 ***

---

Signif. codes: 0 ‘***’ 0.001 ‘**’ 0.01 ‘*’ 0.05 ‘.’ 0.1 ‘ ’ 1

beta 2.5 % 97.5 %

(Intercept) 0.00 0.00 0.00

IRTmax 1.36 1.15 1.65

# univariable logistic regression for M2 yes/no and IRTmax dichotomised by the median

glm(formula = m2bin ~ factor(IRTmax_median), family = "binomial", data = washed)

Deviance Residuals:

Min 1Q Median 3Q Max

-0.4863 -0.4863 -0.2448 -0.2448 2.6543

Coefficients:

Estimate Std. Error z value Pr(>|z|)

(Intercept) -3.4927 0.3589 -9.732 < 2e-16 ***

factor(IRTmax_median)1 1.4172 0.4049 3.500 0.000466 ***

---

Signif. codes: 0 ‘***’ 0.001 ‘**’ 0.01 ‘*’ 0.05 ‘.’ 0.1 ‘ ’ 1

(Dispersion parameter for binomial family taken to be 1)

Null deviance: 287.90 on 557 degrees of freedom

Residual deviance: 272.82 on 556 degrees of freedom

AIC: 276.82

Number of Fisher Scoring iterations: 6

Single term deletions

Model:

m2bin ~ factor(IRTmax_median)

Df Deviance AIC LRT Pr(>Chi)

<none> 272.82 276.82

factor(IRTmax_median) 1 287.90 289.90 15.084 0.0001028 ***

---

Signif. codes: 0 ‘***’ 0.001 ‘**’ 0.01 ‘*’ 0.05 ‘.’ 0.1 ‘ ’ 1

beta 2.5 % 97.5 %

(Intercept) 0.03 0.01 0.06

factor(IRTmax_median)1 4.13 1.96 9.77

# univariable logistic regression M2 yes/no and locomotion score

glm(formula = m2bin ~ factor(locomotion score), family = "binomial", data = washed)

Deviance Residuals:

Min 1Q Median 3Q Max

-0.7090 -0.4157 -0.4157 -0.4157 2.3548

Coefficients:

Estimate Std. Error z value Pr(>|z|)

(Intercept) -2.4054 0.3014 -7.980 1.46e-15 ***

factor(locomotion score)2 0.5596 0.5328 1.050 0.294

factor(locomotion score)3 -0.3026 0.7900 -0.383 0.702

factor(locomotion score)4 1.1527 0.8566 1.346 0.178

---

Signif. codes: 0 ‘***’ 0.001 ‘**’ 0.01 ‘*’ 0.05 ‘.’ 0.1 ‘ ’ 1

(Dispersion parameter for binomial family taken to be 1)

Null deviance: 145.09 on 229 degrees of freedom

Residual deviance: 142.33 on 226 degrees of freedom

(328 observations deleted due to missingness)

AIC: 150.33

Number of Fisher Scoring iterations: 5

Single term deletions

Model:

m2bin ~ factor(locomotion score)

Df Deviance AIC LRT Pr(>Chi)

<none> 142.33 150.33

factor(locomotion score) 3 145.09 147.09 2.7642 0.4294

beta 2.5 % 97.5 %

(Intercept) 0.09 0.05 0.16

factor(locomotion score)2 1.75 0.58 4.83

factor(locomotion score)3 0.74 0.11 2.90

factor(locomotion score)4 3.17 0.44 14.97

# univariable logistic regression M2 yes/no and lame (locomotion score 3+4+5)

glm(formula = m2bin ~ factor(lame), family = "binomial", data = washed)

Deviance Residuals:

Min 1Q Median 3Q Max

-0.4531 -0.4474 -0.4474 -0.4474 2.1686

Coefficients:

Estimate Std. Error z value Pr(>|z|)

(Intercept) -2.25129 0.24780 -9.085 <2e-16 ***

factor(lame)1 0.02667 0.58175 0.046 0.963

---

Signif. codes: 0 ‘***’ 0.001 ‘**’ 0.01 ‘*’ 0.05 ‘.’ 0.1 ‘ ’ 1

(Dispersion parameter for binomial family taken to be 1)

Null deviance: 145.09 on 229 degrees of freedom

Residual deviance: 145.09 on 228 degrees of freedom

(328 observations deleted due to missingness)

AIC: 149.09

Number of Fisher Scoring iterations: 5

Single term deletions

Model:

m2bin ~ factor(lame)

Df Deviance AIC LRT Pr(>Chi)

<none> 145.09 149.09

factor(lame) 1 145.09 147.09 0.0020919 0.9635

beta 2.5 % 97.5 %

(Intercept) 0.11 0.06 0.17

factor(lame)1 1.03 0.28 2.95

# univariable logistic regression M2 yes/no and cleanliness score

glm(formula = m2bin ~ factor(cleanliness score), family = "binomial", data = washed)

Deviance Residuals:

Min 1Q Median 3Q Max

-0.6189 -0.6189 -0.2619 -0.2619 2.6038

Coefficients:

Estimate Std. Error z value Pr(>|z|)

(Intercept) -2.3979 0.7385 -3.247 0.00117 **

factor(cleanliness score)2 -0.9578 0.8473 -1.130 0.25829

factor(cleanliness score)3 0.8425 0.7805 1.079 0.28039

factor(cleanliness score)4 -14.1682 1073.1093 -0.013 0.98947

---

Signif. codes: 0 ‘***’ 0.001 ‘**’ 0.01 ‘*’ 0.05 ‘.’ 0.1 ‘ ’ 1

(Dispersion parameter for binomial family taken to be 1)

Null deviance: 184.46 on 315 degrees of freedom

Residual deviance: 167.10 on 312 degrees of freedom

(242 observations deleted due to missingness)

AIC: 175.1

Number of Fisher Scoring iterations: 15

Single term deletions

Model:

m2bin ~ factor(cleanliness score)

Df Deviance AIC LRT Pr(>Chi)

<none> 167.10 175.10

factor(cleanliness score) 3 184.46 186.46 17.356 0.000597 ***

---

Signif. codes: 0 ‘***’ 0.001 ‘**’ 0.01 ‘*’ 0.05 ‘.’ 0.1 ‘ ’ 1

beta 2.5 % 97.5 %

(Intercept) 0.09 0.01 3.100000e-01

factor(cleanliness score)2 0.38 0.08 2.730000e+00

factor(cleanliness score)3 2.32 0.61 1.526000e+01

factor(cleanliness score)4 0.00 0.00 6.866757e+18

# univariable logistic regression M2 yes/no and dried manure (cleanliness score 3+4)

glm(formula = m2bin ~ factor(dried manure), family = "binomial", data = washed)

Deviance Residuals:

Min 1Q Median 3Q Max

-0.6039 -0.6039 -0.2843 -0.2843 2.5412

Coefficients:

Estimate Std. Error z value Pr(>|z|)

(Intercept) -3.1884 0.3608 -8.838 < 2e-16 ***

factor(dried manure)1 1.5790 0.4397 3.591 0.000329 ***

---

Signif. codes: 0 ‘***’ 0.001 ‘**’ 0.01 ‘*’ 0.05 ‘.’ 0.1 ‘ ’ 1

(Dispersion parameter for binomial family taken to be 1)

Null deviance: 184.46 on 315 degrees of freedom

Residual deviance: 170.07 on 314 degrees of freedom

(242 observations deleted due to missingness)

AIC: 174.07

Number of Fisher Scoring iterations: 6

Single term deletions

Model:

m2bin ~ factor(dried manure)

Df Deviance AIC LRT Pr(>Chi)

<none> 170.07 174.07

factor(dried manure) 1 184.46 186.46 14.391 0.0001485 ***

---

Signif. codes: 0 ‘***’ 0.001 ‘**’ 0.01 ‘*’ 0.05 ‘.’ 0.1 ‘ ’ 1

beta 2.5 % 97.5 %

(Intercept) 0.04 0.02 0.08

factor(dried manure)1 4.85 2.12 12.13

# univariable logistic regression M2 yes/no and farm

glm(formula = m2bin ~ factor(farm), family = "binomial", data = washed)

Deviance Residuals:

Min 1Q Median 3Q Max

-0.5065 -0.4952 -0.3196 -0.3196 2.5476

Coefficients:

Estimate Std. Error z value Pr(>|z|)

(Intercept) -3.2055 0.5888 -5.444 5.2e-08 ***

factor(farm)2 1.2165 0.6588 1.846 0.0648 .

factor(farm)3 1.1686 0.6872 1.700 0.0890 .

factor(farm)4 0.3722 0.8364 0.445 0.6563

factor(farm)5 0.2566 0.6590 0.389 0.6970

---

Signif. codes: 0 ‘***’ 0.001 ‘**’ 0.01 ‘*’ 0.05 ‘.’ 0.1 ‘ ’ 1

(Dispersion parameter for binomial family taken to be 1)

Null deviance: 287.90 on 557 degrees of freedom

Residual deviance: 279.12 on 553 degrees of freedom

AIC: 289.12

Number of Fisher Scoring iterations: 5

Single term deletions

Model:

m2bin ~ factor(farm)

Df Deviance AIC LRT Pr(>Chi)

<none> 279.12 289.12

factor(farm) 4 287.90 289.90 8.7802 0.06683 .

---

Signif. codes: 0 ‘***’ 0.001 ‘**’ 0.01 ‘*’ 0.05 ‘.’ 0.1 ‘ ’ 1

beta 2.5 % 97.5 %

(Intercept) 0.04 0.01 0.11

factor(farm)2 3.38 1.04 15.11

factor(farm)3 3.22 0.92 14.95

factor(farm)4 1.45 0.26 8.11

factor(farm)5 1.29 0.40 5.78

# MULTIVARIABLE LOGISTIC REGRESSION ANALYSES

# UNWASHED FEET DATASET

# full model multivariable logistic regression analysis with M2 yes/no as dependent variable, IRTmax, lame (locomotion score 3+4+5), and dried manure (cleanliness score 3+4) as independent variables, and farm as fixed effect

glm(formula = m2bin ~ IRTmax + factor(lame) + factor(dried manure) + factor(farm), family = "binomial", data = unwashed)

Deviance Residuals:

Min 1Q Median 3Q Max

-1.11976 -0.44803 -0.12645 -0.00004 2.49683

Coefficients:

Estimate Std. Error z value Pr(>|z|)

(Intercept) -38.7311 2788.6264 -0.014 0.98892

IRTmax 0.5882 0.2068 2.845 0.00445 **

factor(lame)1 -0.7892 1.1333 -0.696 0.48621

factor(dried manure)1 0.9349 0.5659 1.652 0.09851 .

factor(farm)2 17.4261 2788.6188 0.006 0.99501

factor(farm)3 18.3847 2788.6188 0.007 0.99474

factor(farm)4 -0.7606 3904.8565 0.000 0.99984

---

Signif. codes: 0 ‘***’ 0.001 ‘**’ 0.01 ‘*’ 0.05 ‘.’ 0.1 ‘ ’ 1

(Dispersion parameter for binomial family taken to be 1)

Null deviance: 117.203 on 204 degrees of freedom

Residual deviance: 84.536 on 198 degrees of freedom

(324 observations deleted due to missingness)

AIC: 98.536

Number of Fisher Scoring iterations: 19

Single term deletions

Model:

m2bin ~ IRTmax + factor(lame) + factor(dried manure) + factor(farm)

Df Deviance AIC LRT Pr(>Chi)

<none> 84.536 98.536

IRTmax 1 96.747 108.747 12.2103 0.0004753 ***

factor(lame) 1 85.101 97.101 0.5649 0.4522906

factor(dried manure) 1 87.287 99.287 2.7509 0.0971968 .

factor(farm) 3 96.992 104.992 12.4553 0.0059756 **

---

Signif. codes: 0 ‘***’ 0.001 ‘**’ 0.01 ‘*’ 0.05 ‘.’ 0.1 ‘ ’ 1

beta 2.5 % 97.5 %

(Intercept) 0.00 NA 3.271980e+60

IRTmax 1.80 1.25 2.810000e+00

factor(lame)1 0.45 0.02 2.980000e+00

factor(dried manure)1 2.55 0.84 7.980000e+00

factor(farm)2 36986741.14 0.00 NA

factor(farm)3 96465326.34 0.00 NA

factor(farm)4 0.47 0.00 3.206777e+40

# final reduced model multivariable logistic regression analysis with M2 yes/no as dependent variable, IRTmax and dried manure (cleanliness score 3+4) as independent variables, and farm as fixed effect

glm(formula = m2bin ~ IRTmax + factor(dried manure) + factor(farm), family = "binomial", data = unwashed)

Deviance Residuals:

Min 1Q Median 3Q Max

-1.0130 -0.4210 -0.2649 -0.1280 2.6119

Coefficients:

Estimate Std. Error z value Pr(>|z|)

(Intercept) -18.4876 4.7666 -3.879 0.000105 ***

IRTmax 0.4643 0.1489 3.119 0.001817 **

factor(dried manure)1 1.3991 0.4768 2.934 0.003342 **

factor(farm)2 0.7184 0.8216 0.874 0.381928

factor(farm)3 1.7096 0.8643 1.978 0.047922 *

factor(farm)4 -0.8541 1.2763 -0.669 0.503373

---

Signif. codes: 0 ‘***’ 0.001 ‘**’ 0.01 ‘*’ 0.05 ‘.’ 0.1 ‘ ’ 1

(Dispersion parameter for binomial family taken to be 1)

Null deviance: 161.04 on 291 degrees of freedom

Residual deviance: 130.43 on 286 degrees of freedom

(237 observations deleted due to missingness)

AIC: 142.43

Number of Fisher Scoring iterations: 7

Single term deletions

Model:

m2bin ~ IRTmax + factor(dried manure) + factor(farm)

Df Deviance AIC LRT Pr(>Chi)

<none> 130.43 142.43

IRTmax 1 143.99 153.99 13.5652 0.0002304 ***

factor(dried manure) 1 139.37 149.37 8.9457 0.0027813 **

factor(farm) 3 139.23 145.23 8.8005 0.0320645 *

---

Signif. codes: 0 ‘***’ 0.001 ‘**’ 0.01 ‘*’ 0.05 ‘.’ 0.1 ‘ ’ 1

beta 2.5 % 97.5 %

(Intercept) 0.00 0.00 0.00

IRTmax 1.59 1.22 2.19

factor(dried manure)1 4.05 1.62 10.70

factor(farm)2 2.05 0.48 14.20

factor(farm)3 5.53 1.17 40.50

factor(farm)4 0.43 0.02 4.86

# full model multivariable logistic regression analysis with M2 yes/no as dependent variable, IRTmax dichotomised by the median, lame (locomotion score 3+4+5), and dried manure (cleanliness score 3+4) as independent variables, and farm as fixed effect

glm(formula = m2bin ~ factor(IRTmax_median) + factor(lame) + factor(dried manure) + factor(farm), family = "binomial", data = unwashed)

Deviance Residuals:

Min 1Q Median 3Q Max

-0.90908 -0.49970 -0.00004 0.00000 2.06999

Coefficients:

Estimate Std. Error z value Pr(>|z|)

(Intercept) -40.4463 4889.6732 -0.008 0.9934

factor(IRTmax_median)1 20.2108 2601.0161 0.008 0.9938

factor(lame)1 -1.0005 1.1604 -0.862 0.3886

factor(dried manure)1 1.0604 0.5847 1.814 0.0697 .

factor(farm)2 18.2179 4140.4856 0.004 0.9965

factor(farm)3 19.5056 4140.4856 0.005 0.9962

factor(farm)4 -0.5941 5966.2769 0.000 0.9999

---

Signif. codes: 0 ‘***’ 0.001 ‘**’ 0.01 ‘*’ 0.05 ‘.’ 0.1 ‘ ’ 1

(Dispersion parameter for binomial family taken to be 1)

Null deviance: 117.203 on 204 degrees of freedom

Residual deviance: 74.871 on 198 degrees of freedom

(324 observations deleted due to missingness)

AIC: 88.871

Number of Fisher Scoring iterations: 20

Single term deletions

Model:

m2bin ~ factor(IRTmax_median) + factor(lame) + factor(dried manure) + factor(farm)

Df Deviance AIC LRT Pr(>Chi)

<none> 74.871 88.871

factor(IRTmax_median) 1 96.747 108.747 21.875 2.91e-06 ***

factor(lame) 1 75.760 87.760 0.889 0.345755

factor(dried manure) 1 78.269 90.269 3.398 0.065277 .

factor(farm) 3 88.100 96.100 13.229 0.004167 **

---

Signif. codes: 0 ‘***’ 0.001 ‘**’ 0.01 ‘*’ 0.05 ‘.’ 0.1 ‘ ’ 1

beta 2.5 % 97.5 %

(Intercept) 0.00 0.00 5.133435e+85

factor(IRTmax_median)1 598991306.77 0.00 NA

factor(lame)1 0.37 0.02 2.560000e+00

factor(dried manure)1 2.89 0.94 9.540000e+00

factor(farm)2 81647820.94 0.00 NA

factor(farm)3 295909500.91 0.00 NA

factor(farm)4 0.55 0.00 7.320380e+72

# final reduced model multivariable logistic regression analysis with M2 yes/no as dependent variable, IRTmax dichotomised by the median and dried manure (cleanliness score 3+4) as independent variables, and farm as fixed effect

glm(formula = m2bin ~ factor(IRTmax_median) + factor(dried manure) + factor(farm), family = "binomial", data = unwashed)

Deviance Residuals:

Min 1Q Median 3Q Max

-1.1771 -0.4061 -0.1935 -0.1111 3.0008

Coefficients:

Estimate Std. Error z value Pr(>|z|)

(Intercept) -5.8880 1.0835 -5.434 5.5e-08 ***

factor(IRTmax_median)1 2.6304 0.8133 3.234 0.00122 **

factor(dried manure)1 1.3969 0.4795 2.913 0.00358 **

factor(farm)2 0.8037 0.8092 0.993 0.32067

factor(farm)3 1.8601 0.8851 2.102 0.03558 *

factor(farm)4 -0.7109 1.2696 -0.560 0.57550

---

Signif. codes: 0 ‘***’ 0.001 ‘**’ 0.01 ‘*’ 0.05 ‘.’ 0.1 ‘ ’ 1

(Dispersion parameter for binomial family taken to be 1)

Null deviance: 161.04 on 291 degrees of freedom

Residual deviance: 127.88 on 286 degrees of freedom

(237 observations deleted due to missingness)

AIC: 139.88

Number of Fisher Scoring iterations: 7

Single term deletions

Model:

m2bin ~ factor(IRTmax_median) + factor(dried manure) + factor(farm)

Df Deviance AIC LRT Pr(>Chi)

<none> 127.88 139.88

factor(IRTmax_median) 1 143.99 153.99 16.1168 5.955e-05 ***

factor(dried manure) 1 136.72 146.72 8.8428 0.002943 **

factor(farm) 3 137.01 143.01 9.1354 0.027544 *

---

Signif. codes: 0 ‘***’ 0.001 ‘**’ 0.01 ‘*’ 0.05 ‘.’ 0.1 ‘ ’ 1

beta 2.5 % 97.5 %

(Intercept) 0.00 0.00 0.02

factor(IRTmax_median)1 13.88 3.41 95.67

factor(dried manure)1 4.04 1.61 10.75

factor(farm)2 2.23 0.54 15.22

factor(farm)3 6.42 1.31 48.71

factor(farm)4 0.49 0.02 5.57

# WASHED FEET DATASET

# full model multivariable logistic regression analysis with M2 yes/no as dependent variable, IRTmax, lame (locomotion score 3+4+5), and dried manure (cleanliness score 3+4) as independent variables, and farm as fixed effect

glm(formula = m2bin ~ IRTmax + factor(lame) + factor(dried manure) + factor(farm), family = "binomial", data = washed)

Deviance Residuals:

Min 1Q Median 3Q Max

-1.3374 -0.3967 -0.2947 -0.1459 2.6720

Coefficients:

Estimate Std. Error z value Pr(>|z|)

(Intercept) -13.3427 3.9344 -3.391 0.000696 ***

IRTmax 0.2605 0.1173 2.221 0.026374 *

factor(lame)1 0.2003 0.7214 0.278 0.781243

factor(dried manure)1 1.6124 0.5368 3.004 0.002668 **

factor(farm)2 2.1418 1.1079 1.933 0.053203 .

factor(farm)3 2.9543 1.1501 2.569 0.010205 *

factor(farm)4 1.3059 1.2734 1.026 0.305111

---

Signif. codes: 0 ‘***’ 0.001 ‘**’ 0.01 ‘*’ 0.05 ‘.’ 0.1 ‘ ’ 1

(Dispersion parameter for binomial family taken to be 1)

Null deviance: 140.36 on 228 degrees of freedom

Residual deviance: 112.33 on 222 degrees of freedom

(329 observations deleted due to missingness)

AIC: 126.33

Number of Fisher Scoring iterations: 6

Single term deletions

Model:

m2bin ~ IRTmax + factor(lame) + factor(dried manure) + factor(farm)

Df Deviance AIC LRT Pr(>Chi)

<none> 112.33 126.33

IRTmax 1 118.88 130.88 6.5439 0.010524 *

factor(lame) 1 112.41 124.41 0.0752 0.783912

factor(dried manure) 1 122.32 134.32 9.9856 0.001578 **

factor(farm) 3 123.88 131.88 11.5439 0.009121 **

---

Signif. codes: 0 ‘***’ 0.001 ‘**’ 0.01 ‘*’ 0.05 ‘.’ 0.1 ‘ ’ 1

beta 2.5 % 97.5 %

(Intercept) 0.00 0.00 0.00

IRTmax 1.30 1.06 1.68

factor(lame)1 1.22 0.25 4.62

factor(dried manure)1 5.01 1.82 15.43

factor(farm)2 8.51 1.40 166.52

factor(farm)3 19.19 2.83 392.91

factor(farm)4 3.69 0.32 84.18

# final reduced model multivariable logistic regression analysis with M2 yes/no as dependent variable, IRTmax and dried manure (cleanliness score 3+4) as independent variables, and farm as fixed effect

glm(formula = m2bin ~ IRTmax + factor(dried manure) + factor(farm), family = "binomial", data = washed)

Deviance Residuals:

Min 1Q Median 3Q Max

-1.4450 -0.4135 -0.2891 -0.1735 2.7565

Coefficients:

Estimate Std. Error z value Pr(>|z|)

(Intercept) -13.8538 3.6105 -3.837 0.000124 ***

IRTmax 0.2968 0.1091 2.721 0.006511 **

factor(dried manure)1 1.6716 0.4688 3.566 0.000363 ***

factor(farm)2 1.3502 0.6815 1.981 0.047568 *

factor(farm)3 2.3732 0.7575 3.133 0.001729 **

factor(farm)4 0.6652 0.8624 0.771 0.440461

---

Signif. codes: 0 ‘***’ 0.001 ‘**’ 0.01 ‘*’ 0.05 ‘.’ 0.1 ‘ ’ 1

(Dispersion parameter for binomial family taken to be 1)

Null deviance: 184.46 on 315 degrees of freedom

Residual deviance: 151.40 on 310 degrees of freedom

(242 observations deleted due to missingness)

AIC: 163.4

Number of Fisher Scoring iterations: 6

Single term deletions

Model:

m2bin ~ IRTmax + factor(dried manure) + factor(farm)

Df Deviance AIC LRT Pr(>Chi)

<none> 151.40 163.40

IRTmax 1 161.35 171.35 9.9551 0.0016041 **

factor(dried manure) 1 165.58 175.58 14.1876 0.0001655 ***

factor(farm) 3 164.34 170.34 12.9421 0.0047636 **

---

Signif. codes: 0 ‘***’ 0.001 ‘**’ 0.01 ‘*’ 0.05 ‘.’ 0.1 ‘ ’ 1

beta 2.5 % 97.5 %

(Intercept) 0.00 0.00 0.00

IRTmax 1.35 1.11 1.70

factor(dried manure)1 5.32 2.20 14.07

factor(farm)2 3.86 1.13 17.85

factor(farm)3 10.73 2.66 56.01

factor(farm)4 1.94 0.33 11.38

# full model multivariable logistic regression analysis with M2 yes/no as dependent variable, IRTmax dichotomised by the median, lame (locomotion score 3+4+5), and dried manure (cleanliness score 3+4) as independent variables, and farm as fixed effect

glm(formula = m2bin ~ factor(IRTmax_median) + factor(lame) + factor(dried manure) + factor(farm), family = "binomial", data = washed)

Deviance Residuals:

Min 1Q Median 3Q Max

-1.1554 -0.3906 -0.2678 -0.1376 2.8389

Coefficients:

Estimate Std. Error z value Pr(>|z|)

(Intercept) -6.1325 1.2526 -4.896 9.8e-07 ***

factor(IRTmax_median)1 1.4765 0.6389 2.311 0.02082 *

factor(lame)1 0.1361 0.7370 0.185 0.85351

factor(dried manure)1 1.6458 0.5301 3.105 0.00191 **

factor(farm)2 2.1209 1.1094 1.912 0.05590 .

factor(farm)3 2.8221 1.1491 2.456 0.01405 *

factor(farm)4 1.3102 1.2735 1.029 0.30356

---

Signif. codes: 0 ‘***’ 0.001 ‘**’ 0.01 ‘*’ 0.05 ‘.’ 0.1 ‘ ’ 1

(Dispersion parameter for binomial family taken to be 1)

Null deviance: 140.36 on 228 degrees of freedom

Residual deviance: 112.63 on 222 degrees of freedom

(329 observations deleted due to missingness)

AIC: 126.63

Number of Fisher Scoring iterations: 6

Single term deletions

Model:

m2bin ~ factor(IRTmax_median) + factor(lame) + factor(dried manure) + factor(farm)

Df Deviance AIC LRT Pr(>Chi)

<none> 112.63 126.63

factor(IRTmax_median) 1 118.88 130.88 6.2493 0.012424 *

factor(lame) 1 112.66 124.66 0.0336 0.854647

factor(dried manure) 1 123.36 135.36 10.7286 0.001055 **

factor(farm) 3 123.09 131.09 10.4609 0.015029 *

---

Signif. codes: 0 ‘***’ 0.001 ‘**’ 0.01 ‘*’ 0.05 ‘.’ 0.1 ‘ ’ 1

beta 2.5 % 97.5 %

(Intercept) 0.00 0.00 0.02

factor(IRTmax_median)1 4.38 1.35 17.43

factor(lame)1 1.15 0.23 4.48

factor(dried manure)1 5.19 1.91 15.76

factor(farm)2 8.34 1.37 163.36

factor(farm)3 16.81 2.49 344.10

factor(farm)4 3.71 0.32 84.56

# final reduced model multivariable logistic regression analysis with M2 yes/no as dependent variable, IRTmax dichotomised by the median and dried manure (cleanliness score 3+4) as independent variables, and farm as fixed effect

glm(formula = m2bin ~ factor(IRTmax_median) + factor(dried manure) + factor(farm), family = "binomial", data = washed)

Deviance Residuals:

Min 1Q Median 3Q Max

-1.1440 -0.3980 -0.2626 -0.1881 2.9055

Coefficients:

Estimate Std. Error z value Pr(>|z|)

(Intercept) -5.5870 0.8604 -6.494 8.39e-11 ***

factor(IRTmax_median)1 1.5611 0.5583 2.796 0.005174 **

factor(dried manure)1 1.7101 0.4650 3.678 0.000235 ***

factor(farm)2 1.3807 0.6839 2.019 0.043495 *

factor(farm)3 2.2367 0.7519 2.975 0.002933 **

factor(farm)4 0.6699 0.8623 0.777 0.437232

---

Signif. codes: 0 ‘***’ 0.001 ‘**’ 0.01 ‘*’ 0.05 ‘.’ 0.1 ‘ ’ 1

(Dispersion parameter for binomial family taken to be 1)

Null deviance: 184.46 on 315 degrees of freedom

Residual deviance: 151.88 on 310 degrees of freedom

(242 observations deleted due to missingness)

AIC: 163.88

Number of Fisher Scoring iterations: 6

Single term deletions

Model:

m2bin ~ factor(IRTmax_median) + factor(dried manure) + factor(farm)

Df Deviance AIC LRT Pr(>Chi)

<none> 151.88 163.88

factor(IRTmax_median) 1 161.35 171.35 9.4672 0.0020918 **

factor(dried manure) 1 167.01 177.01 15.1262 0.0001006 ***

factor(farm) 3 163.68 169.68 11.7935 0.0081249 **

---

Signif. codes: 0 ‘***’ 0.001 ‘**’ 0.01 ‘*’ 0.05 ‘.’ 0.1 ‘ ’ 1

beta 2.5 % 97.5 %

(Intercept) 0.00 0.00 0.02

factor(IRTmax_median)1 4.76 1.71 15.83

factor(dried manure)1 5.53 2.30 14.51

factor(farm)2 3.98 1.16 18.47

factor(farm)3 9.36 2.35 48.40

factor(farm)4 1.95 0.33 11.43
