# Supplementary material for: Broad-spectrum infrared thermography for detection of M2 digital dermatitis lesions on hind feet of standing dairy cattle
Source: PLoS One. 2023 Jan 17;18(1):e0280098. doi: 10.1371/journal.pone.0280098 (PMC9844892; doi:10.1371/journal.pone.0280098)
Supplement: S2 File — Full results of all regression analyses performed investigating the association between maximum infrared temperature (IRTmax) of the plantar pastern region of feet from standing dairy cattle and the presence of any lesions of digital dermatitis. (DOCX) [file pone.0280098.s003.docx]

**S3 Regression analyses M2 lesions. Full results of all regression analyses performed investigating the association between maximum infrared temperature (IRTmax) of the plantar pastern region of feet from standing dairy cattle and the presence of any lesions of digital dermatitis.**

# UNIVARIABLE LOGISTIC REGRESSION ANALYSES

# UNWASHED FEET DATASET

# univariable logistic regression for DD yes/no and IRTmax

glm(formula = DDbin ~ IRTmax, family = "binomial", data = unwashed)

Deviance Residuals:

Min 1Q Median 3Q Max

-1.6285 -1.2735 0.8548 1.0044 1.7627

Coefficients:

Estimate Std. Error z value Pr(>|z|)

(Intercept) -4.67534 1.04839 -4.460 8.21e-06 ***

IRTmax 0.16549 0.03442 4.807 1.53e-06 ***

---

Signif. codes: 0 ‘***’ 0.001 ‘**’ 0.01 ‘*’ 0.05 ‘.’ 0.1 ‘ ’ 1

(Dispersion parameter for binomial family taken to be 1)

Null deviance: 717.62 on 528 degrees of freedom

Residual deviance: 692.62 on 527 degrees of freedom

AIC: 696.62

Number of Fisher Scoring iterations: 4

Single term deletions

Model:

DDbin ~ IRTmax

Df Deviance AIC LRT Pr(>Chi)

<none> 692.62 696.62

IRTmax 1 717.62 719.62 24.995 5.747e-07 ***

---

Signif. codes: 0 ‘***’ 0.001 ‘**’ 0.01 ‘*’ 0.05 ‘.’ 0.1 ‘ ’ 1

beta 2.5 % 97.5 %

(Intercept) 0.01 0.0 0.07

IRTmax 1.18 1.1 1.26

# univariable logistic regression for DD yes/no and IRTmax dichotomised by the median

glm(formula = DDbin ~ factor(IRTmax_median), family = "binomial", data = unwashed)

Deviance Residuals:

Min 1Q Median 3Q Max

-1.4546 -1.2098 0.9235 1.1454 1.1454

Coefficients:

Estimate Std. Error z value Pr(>|z|)

(Intercept) 0.07579 0.12318 0.615 0.53835

factor(IRTmax_median)1 0.55571 0.17839 3.115 0.00184 **

---

Signif. codes: 0 ‘***’ 0.001 ‘**’ 0.01 ‘*’ 0.05 ‘.’ 0.1 ‘ ’ 1

(Dispersion parameter for binomial family taken to be 1)

Null deviance: 717.62 on 528 degrees of freedom

Residual deviance: 707.81 on 527 degrees of freedom

AIC: 711.81

Number of Fisher Scoring iterations: 4

Single term deletions

Model:

DDbin ~ factor(IRTmax_median)

Df Deviance AIC LRT Pr(>Chi)

<none> 707.81 711.81

factor(IRTmax_median) 1 717.62 719.62 9.8059 0.00174 **

---

Signif. codes: 0 ‘***’ 0.001 ‘**’ 0.01 ‘*’ 0.05 ‘.’ 0.1 ‘ ’ 1

beta 2.5 % 97.5 %

(Intercept) 1.08 0.85 1.37

factor(IRTmax_median)1 1.74 1.23 2.48

# univariable logistic regression DD yes/no and locomotion score

glm(formula = DDbin ~ factor(locomotion score), family = "binomial", data = unwashed)

Deviance Residuals:

Min 1Q Median 3Q Max

-1.7080 -1.4722 0.9005 0.9089 1.1774

Coefficients:

Estimate Std. Error z value Pr(>|z|)

(Intercept) 0.67067 0.18326 3.660 0.000253 ***

factor(locomotion score)2 0.52325 0.40483 1.293 0.196182

factor(locomotion score)3 0.02247 0.49787 0.045 0.963997

factor(locomotion score)4 -0.67067 0.73047 -0.918 0.358545

factor(locomotion score)5 13.89539 882.74339 0.016 0.987441

---

Signif. codes: 0 ‘***’ 0.001 ‘**’ 0.01 ‘*’ 0.05 ‘.’ 0.1 ‘ ’ 1

(Dispersion parameter for binomial family taken to be 1)

Null deviance: 258.39 on 205 degrees of freedom

Residual deviance: 254.69 on 201 degrees of freedom

(323 observations deleted due to missingness)

AIC: 264.69

Number of Fisher Scoring iterations: 13

Single term deletions

Model:

DDbin ~ factor(locomotion score)

Df Deviance AIC LRT Pr(>Chi)

<none> 254.69 264.69

factor(locomotion score) 4 258.39 260.39 3.7028 0.4477

beta 2.5 % 97.5 %

(Intercept) 1.96 1.37 2.82

factor(locomotion score)2 1.69 0.78 3.89

factor(locomotion score)3 1.02 0.40 2.86

factor(locomotion score)4 0.51 0.12 2.25

factor(locomotion score)5 1083160.22 0.00 NA

# univariable logistic regression DD yes/no and lame (locomotion score 3+4+5)

glm(formula = DDbin ~ factor(lame), family = "binomial", data = unwashed)

Deviance Residuals:

Min 1Q Median 3Q Max

-1.5252 -1.5252 0.8657 0.8657 0.9558

Coefficients:

Estimate Std. Error z value Pr(>|z|)

(Intercept) 0.7885 0.1626 4.848 1.24e-06 ***

factor(lame)1 -0.2419 0.4123 -0.587 0.557

---

Signif. codes: 0 ‘***’ 0.001 ‘**’ 0.01 ‘*’ 0.05 ‘.’ 0.1 ‘ ’ 1

(Dispersion parameter for binomial family taken to be 1)

Null deviance: 258.39 on 205 degrees of freedom

Residual deviance: 258.05 on 204 degrees of freedom

(323 observations deleted due to missingness)

AIC: 262.05

Number of Fisher Scoring iterations: 4

Single term deletions

Model:

DDbin ~ factor(lame)

Df Deviance AIC LRT Pr(>Chi)

<none> 258.05 262.05

factor(lame) 1 258.39 260.39 0.33881 0.5605

beta 2.5 % 97.5 %

(Intercept) 2.20 1.61 3.05

factor(lame)1 0.79 0.35 1.81

# univariable logistic regression DD yes/no and cleanliness score

glm(formula = DDbin ~ factor(cleanliness score), family = "binomial", data = unwashed)

Deviance Residuals:

Min 1Q Median 3Q Max

-1.6033 -1.4107 0.8047 0.9608 1.0579

Coefficients:

Estimate Std. Error z value Pr(>|z|)

(Intercept) 0.47000 0.40311 1.166 0.244

factor(cleanliness score)2 0.06351 0.43418 0.146 0.884

factor(cleanliness score)3 0.49141 0.46440 1.058 0.290

factor(cleanliness score)4 -0.18232 0.86362 -0.211 0.833

(Dispersion parameter for binomial family taken to be 1)

Null deviance: 375.31 on 291 degrees of freedom

Residual deviance: 372.47 on 288 degrees of freedom

(237 observations deleted due to missingness)

AIC: 380.47

Number of Fisher Scoring iterations: 4

Single term deletions

Model:

DDbin ~ factor(cleanliness score)

Df Deviance AIC LRT Pr(>Chi)

<none> 372.47 380.47

factor(cleanliness score) 3 375.31 377.31 2.8373 0.4174

beta 2.5 % 97.5 %

(Intercept) 1.60 0.74 3.65

factor(cleanliness score)2 1.07 0.44 2.47

factor(cleanliness score)3 1.63 0.64 4.03

factor(cleanliness score)4 0.83 0.15 4.97

# univariable logistic regression DD yes/no and dried manure (cleanliness score 3+4)

glm(formula = DDbin ~ factor(dried manure), family = "binomial", data = unwashed)

Deviance Residuals:

Min 1Q Median 3Q Max

-1.5798 -1.4068 0.8227 0.9641 0.9641

Coefficients:

Estimate Std. Error z value Pr(>|z|)

(Intercept) 0.5248 0.1497 3.505 0.000456 ***

factor(dried manure)1 0.3846 0.2661 1.445 0.148355

---

Signif. codes: 0 ‘***’ 0.001 ‘**’ 0.01 ‘*’ 0.05 ‘.’ 0.1 ‘ ’ 1

(Dispersion parameter for binomial family taken to be 1)

Null deviance: 375.31 on 291 degrees of freedom

Residual deviance: 373.18 on 290 degrees of freedom

(237 observations deleted due to missingness)

AIC: 377.18

Number of Fisher Scoring iterations: 4

Single term deletions

Model:

DDbin ~ factor(dried manure)

Df Deviance AIC LRT Pr(>Chi)

<none> 373.18 377.18

factor(dried manure) 1 375.31 377.31 2.1308 0.1444

beta 2.5 % 97.5 %

(Intercept) 1.69 1.26 2.28

factor(dried manure)1 1.47 0.88 2.50

# univariable logistic regression DD yes/no and farm

glm(formula = DDbin ~ factor(farm), family = "binomial", data = unwashed)

Deviance Residuals:

Min 1Q Median 3Q Max

-1.692 -1.170 0.739 1.177 1.185

Coefficients:

Estimate Std. Error z value Pr(>|z|)

(Intercept) 0.5465 0.2679 2.040 0.0413 *

factor(farm)2 0.6120 0.3470 1.763 0.0778 .

factor(farm)3 -0.5465 0.3568 -1.532 0.1256

factor(farm)4 0.2419 0.4108 0.589 0.5559

factor(farm)5 -0.5635 0.2979 -1.892 0.0585 .

---

Signif. codes: 0 ‘***’ 0.001 ‘**’ 0.01 ‘*’ 0.05 ‘.’ 0.1 ‘ ’ 1

(Dispersion parameter for binomial family taken to be 1)

Null deviance: 717.62 on 528 degrees of freedom

Residual deviance: 689.71 on 524 degrees of freedom

AIC: 699.71

Number of Fisher Scoring iterations: 4

Single term deletions

Model:

DDbin ~ factor(farm)

Df Deviance AIC LRT Pr(>Chi)

<none> 689.71 699.71

factor(farm) 4 717.62 719.62 27.906 1.303e-05 ***

---

Signif. codes: 0 ‘***’ 0.001 ‘**’ 0.01 ‘*’ 0.05 ‘.’ 0.1 ‘ ’ 1

beta 2.5 % 97.5 %

(Intercept) 1.73 1.03 2.97

factor(farm)2 1.84 0.93 3.65

factor(farm)3 0.58 0.29 1.16

factor(farm)4 1.27 0.57 2.88

factor(farm)5 0.57 0.31 1.01

# WASHED FEET DATASET

# univariable logistic regression for DD yes/no and IRTmax

glm(formula = DDbin ~ IRTmax, family = "binomial", data = washed)

Deviance Residuals:

Min 1Q Median 3Q Max

-1.6157 -1.3014 0.8829 0.9965 1.7233

Coefficients:

Estimate Std. Error z value Pr(>|z|)

(Intercept) -3.8442 0.9569 -4.017 5.89e-05 ***

IRTmax 0.1377 0.0312 4.413 1.02e-05 ***

---

Signif. codes: 0 ‘***’ 0.001 ‘**’ 0.01 ‘*’ 0.05 ‘.’ 0.1 ‘ ’ 1

(Dispersion parameter for binomial family taken to be 1)

Null deviance: 755.53 on 557 degrees of freedom

Residual deviance: 734.84 on 556 degrees of freedom

AIC: 738.84

Number of Fisher Scoring iterations: 4

Single term deletions

Model:

DDbin ~ IRTmax

Df Deviance AIC LRT Pr(>Chi)

<none> 734.84 738.84

IRTmax 1 755.53 757.53 20.696 5.382e-06 ***

---

Signif. codes: 0 ‘***’ 0.001 ‘**’ 0.01 ‘*’ 0.05 ‘.’ 0.1 ‘ ’ 1

beta 2.5 % 97.5 %

(Intercept) 0.02 0.00 0.14

IRTmax 1.15 1.08 1.22

# univariable logistic regression for DD yes/no and IRTmax dichotomised by the median

glm(formula = DDbin ~ factor(IRTmax_median), family = "binomial", data = washed)

Deviance Residuals:

Min 1Q Median 3Q Max

-1.4047 -1.2634 0.9659 1.0938 1.0938

Coefficients:

Estimate Std. Error z value Pr(>|z|)

(Intercept) 0.1999 0.1221 1.637 0.1015

factor(IRTmax_median)1 0.3202 0.1727 1.855 0.0637 .

---

Signif. codes: 0 ‘***’ 0.001 ‘**’ 0.01 ‘*’ 0.05 ‘.’ 0.1 ‘ ’ 1

(Dispersion parameter for binomial family taken to be 1)

Null deviance: 755.53 on 557 degrees of freedom

Residual deviance: 752.08 on 556 degrees of freedom

AIC: 756.08

Number of Fisher Scoring iterations: 4

Single term deletions

Model:

DDbin ~ factor(IRTmax_median)

Df Deviance AIC LRT Pr(>Chi)

<none> 752.08 756.08

factor(IRTmax_median) 1 755.53 757.53 3.4497 0.06326 .

---

Signif. codes: 0 ‘***’ 0.001 ‘**’ 0.01 ‘*’ 0.05 ‘.’ 0.1 ‘ ’ 1

beta 2.5 % 97.5 %

(Intercept) 1.22 0.96 1.55

factor(IRTmax_median)1 1.38 0.98 1.93

# univariable logistic regression DD yes/no and locomotion score

glm(formula = DDbin ~ factor(locomotion score), family = "binomial", data = washed)

Deviance Residuals:

Min 1Q Median 3Q Max

-1.6651 -1.4730 0.9082 0.9082 1.0842

Coefficients:

Estimate Std. Error z value Pr(>|z|)

(Intercept) 0.6725 0.1756 3.831 0.000128 ***

factor(locomotion score)2 0.4261 0.3899 1.093 0.274503

factor(locomotion score)3 -0.0259 0.4115 -0.063 0.949816

factor(locomotion score)4 -0.4494 0.6934 -0.648 0.516937

---

Signif. codes: 0 ‘***’ 0.001 ‘**’ 0.01 ‘*’ 0.05 ‘.’ 0.1 ‘ ’ 1

(Dispersion parameter for binomial family taken to be 1)

Null deviance: 290.43 on 229 degrees of freedom

Residual deviance: 288.53 on 226 degrees of freedom

(328 observations deleted due to missingness)

AIC: 296.53

Number of Fisher Scoring iterations: 4

Single term deletions

Model:

DDbin ~ factor(locomotion score)

Df Deviance AIC LRT Pr(>Chi)

<none> 288.53 296.53

factor(locomotion score) 3 290.43 292.43 1.8977 0.5939

beta 2.5 % 97.5 %

(Intercept) 1.96 1.40 2.78

factor(locomotion score)2 1.53 0.73 3.41

factor(locomotion score)3 0.97 0.44 2.25

factor(locomotion score)4 0.64 0.16 2.68

# univariable logistic regression DD yes/no and lame (locomotion score 3+4+5)

glm(formula = DDbin ~ factor(lame), family = "binomial", data = washed)

Deviance Residuals:

Min 1Q Median 3Q Max

-1.5149 -1.5149 0.8740 0.8740 0.9544

Coefficients:

Estimate Std. Error z value Pr(>|z|)

(Intercept) 0.7655 0.1563 4.899 9.66e-07 ***

factor(lame)1 -0.2154 0.3599 -0.599 0.549

---

Signif. codes: 0 ‘***’ 0.001 ‘**’ 0.01 ‘*’ 0.05 ‘.’ 0.1 ‘ ’ 1

(Dispersion parameter for binomial family taken to be 1)

Null deviance: 290.43 on 229 degrees of freedom

Residual deviance: 290.08 on 228 degrees of freedom

(328 observations deleted due to missingness)

AIC: 249.08

Number of Fisher Scoring iterations: 4

Single term deletions

Model:

DDbin ~ factor(lame)

Df Deviance AIC LRT Pr(>Chi)

<none> 290.08 294.0

factor(lame) 1 290.43 292.43 0.35367 0.552

beta 2.5 % 97.5 %

(Intercept) 2.15 1.59 2.94

factor(lame)1 0.81 0.40 1.66

# univariable logistic regression DD yes/no and cleanliness score

glm(formula = DDbin ~ factor(cleanliness score), family = "binomial", data = washed)

Deviance Residuals:

Min 1Q Median 3Q Max

-1.6651 -1.4194 0.8337 0.9533 1.0108

Coefficients:

Estimate Std. Error z value Pr(>|z|)

(Intercept) 1.0986 0.4714 2.331 0.0198 *

factor(cleanliness score)2 -0.5456 0.4964 -1.099 0.2718

factor(cleanliness score)3 -0.2205 0.5162 -0.427 0.6692

factor(cleanliness score)4 -0.6931 1.0274 -0.675 0.4999

---

Signif. codes: 0 ‘***’ 0.001 ‘**’ 0.01 ‘*’ 0.05 ‘.’ 0.1 ‘ ’ 1

(Dispersion parameter for binomial family taken to be 1)

Null deviance: 401.81 on 315 degrees of freedom

Residual deviance: 399.34 on 312 degrees of freedom

(242 observations deleted due to missingness)

AIC: 407.34

Number of Fisher Scoring iterations: 4

Single term deletions

Model:

DDbin ~ factor(cleanliness score)

Df Deviance AIC LRT Pr(>Chi)

<none> 399.34 407.34

factor(cleanliness score) 3 401.81 403.81 2.4754 0.4798

beta 2.5 % 97.5 %

(Intercept) 3.00 1.26 8.27

factor(cleanliness score)2 0.58 0.20 1.46

factor(cleanliness score)3 0.80 0.27 2.11

factor(cleanliness score)4 0.50 0.07 4.48

# univariable logistic regression DD yes/no and dried manure (cleanliness score 3+4)

glm(formula = DDbin ~ factor(dried manure), family = "binomial", data = washed)

Deviance Residuals:

Min 1Q Median 3Q Max

-1.5555 -1.4461 0.8416 0.9307 0.9307

Coefficients:

Estimate Std. Error z value Pr(>|z|)

(Intercept) 0.6125 0.1474 4.156 3.23e-05 ***

factor(dried manure)1 2431 0.2522 0.964 0.335

---

Signif. codes: 0 ‘***’ 0.001 ‘**’ 0.01 ‘*’ 0.05 ‘.’ 0.1 ‘ ’ 1

(Dispersion parameter for binomial family taken to be 1)

Null deviance: 401.81 on 315 degrees of freedom

Residual deviance: 400.87 on 314 degrees of freedom

(242 observations deleted due to missingness)

AIC: 404.87

Number of Fisher Scoring iterations: 4

Single term deletions

Model:

DDbin ~ factor(dried manure)

Df Deviance AIC LRT Pr(>Chi)

<none> 400.87 404.87

factor(dried manure) 1 401.81 403.81 0.9389 0.3326

beta 2.5 % 97.5 %

(Intercept) 1.85 1.39 2.47

factor(dried manure)1 1.28 0.78 2.11

# univariable logistic regression DD yes/no and farm

glm(formula = DDbin ~ factor(farm), family = "binomial", data = washed)

Deviance Residuals:

Min 1Q Median 3Q Max

-1.7588 -1.1528 0.6921 1.1127 1.2022

Coefficients:

Estimate Std. Error z value Pr(>|z|)

(Intercept) 0.3409 0.2312 1.474 0.14039

factor(farm)2 0.9662 0.3297 2.930 0.00338 **

factor(farm)3 0.9662 0.3297 2.930 0.00338 **

factor(farm)4 0.7089 0.3872 1.831 0.06711 .

factor(farm)5 -0.3990 0.2647 -1.507 0.13173

---

Signif. codes: 0 ‘***’ 0.001 ‘**’ 0.01 ‘*’ 0.05 ‘.’ 0.1 ‘ ’ 1

(Dispersion parameter for binomial family taken to be 1)

Null deviance: 755.53 on 557 degrees of freedom

Residual deviance: 719.77 on 553 degrees of freedom

AIC: 729.77

Number of Fisher Scoring iterations: 4

Single term deletions

Model:

DDbin ~ factor(farm)

Df Deviance AIC LRT Pr(>Chi)

<none> 719.77 729.7

factor(farm) 4 755.53 757.53 35.769 3.228e-07 ***

---

Signif. codes: 0 ‘***’ 0.001 ‘**’ 0.01 ‘*’ 0.05 ‘.’ 0.1 ‘ ’ 1

beta 2.5 % 97.5 %

(Intercept) 1.41 0.90 2.23

factor(farm)2 2.63 1.38 5.06

factor(farm)3 0.83 0.44 1.57

factor(farm)4 2.03 0.96 4.43

factor(farm)5 0.67 0.40 1.12

# MULTIVARIABLE LOGISTIC REGRESSION ANALYSES

# UNWASHED FEET DATASET

# full model multivariable logistic regression analysis with DD yes/no as dependent variable, IRTmax, lame (locomotion score 3+4+5), and dried manure (cleanliness score 3+4) as independent variables, and farm as fixed effect

glm(formula = DDbin ~ IRTmax + factor(lame) + factor(dried manure) + factor(farm), family = "binomial", data = unwashed)

Deviance Residuals:

Min 1Q Median 3Q Max

-2.1990 -1.1515 0.6571 0.8034 1.8964

Coefficients:

Estimate Std. Error z value Pr(>|z|)

(Intercept) -4.26815 2.01931 -2.114 0.03454 *

IRTmax 0.19447 0.06731 2.889 0.00386 **

factor(lame)1 -0.86802 0.48096 -1.805 0.07111 .

factor(dried manure)1 0.24882 0.34320 0.725 0.46845

factor(farm)2 -0.84074 0.55788 -1.507 0.13180

factor(farm)3 -1.29677 0.54119 -2.396 0.01657 *

factor(farm)4 -0.58602 0.60737 -0.965 0.33462

---

Signif. codes: 0 ‘***’ 0.001 ‘**’ 0.01 ‘*’ 0.05 ‘.’ 0.1 ‘ ’ 1

(Dispersion parameter for binomial family taken to be 1)

Null deviance: 257.62 on 204 degrees of freedom

Residual deviance: 233.42 on 198 degrees of freedom

(324 observations deleted due to missingness)

AIC: 247.42

Number of Fisher Scoring iterations: 4

Single term deletions

Model:

DDbin ~ IRTmax + factor(lame) + factor(dried manure) + factor(farm)

Df Deviance AIC LRT Pr(>Chi)

<none> 233.42 247.42

IRTmax 1 242.52 254.52 9.1009 0.002555 **

factor(lame) 1 236.64 248.64 3.2154 0.072949 .

factor(dried manure) 1 233.95 245.95 0.5306 0.466360

factor(farm) 3 240.13 248.13 6.7106 0.081716 .

---

Signif. codes: 0 ‘***’ 0.001 ‘**’ 0.01 ‘*’ 0.05 ‘.’ 0.1 ‘ ’ 1

beta 2.5 % 97.5 %

(Intercept) 0.01 0.00 0.67

IRTmax 1.21 1.07 1.39

factor(lame)1 0.42 0.16 1.09

factor(dried manure)1 1.28 0.66 2.55

factor(farm)2 0.43 0.14 1.24

factor(farm)3 0.27 0.09 0.76

factor(farm)4 0.56 0.16 1.82

# final reduced model multivariable logistic regression analysis with DD yes/no as dependent variable, IRTmax as independent variable, and farm as fixed effect

glm(formula = DDbin ~ IRTmax + factor(farm), family = "binomial", data = unwashed)

Deviance Residuals:

Min 1Q Median 3Q Max

-1.8318 -1.1990 0.7211 1.0499 1.6832

Coefficients:

Estimate Std. Error z value Pr(>|z|)

(Intercept) -3.50848 1.14568 -3.062 0.002196 **

IRTmax 0.13517 0.03719 3.635 0.000278 ***

factor(farm)2 0.32970 0.35840 0.920 0.357613

factor(farm)3 -0.37423 0.36539 -1.024 0.305751

factor(farm)4 0.21905 0.41535 0.527 0.597922

factor(farm)5 -0.59405 0.30179 -1.968 0.049023 *

---

Signif. codes: 0 ‘***’ 0.001 ‘**’ 0.01 ‘*’ 0.05 ‘.’ 0.1 ‘ ’ 1

(Dispersion parameter for binomial family taken to be 1)

Null deviance: 717.62 on 528 degrees of freedom

Residual deviance: 675.71 on 523 degrees of freedom

AIC: 687.71

Number of Fisher Scoring iterations: 4

Single term deletions

Model:

DDbin ~ IRTmax + factor(farm)

Df Deviance AIC LRT Pr(>Chi)

<none> 675.71 687.71

IRTmax 1 689.71 699.71 13.999 0.0001829 ***

factor(farm) 4 692.62 696.62 16.910 0.0020125 **

---

Signif. codes: 0 ‘***’ 0.001 ‘**’ 0.01 ‘*’ 0.05 ‘.’ 0.1 ‘ ’ 1

beta 2.5 % 97.5 %

(Intercept) 0.03 0.00 0.27

IRTmax 1.14 1.07 1.23

factor(farm)2 1.39 0.69 2.81

factor(farm)3 0.69 0.33 1.40

factor(farm)4 1.24 0.55 2.84

factor(farm)5 0.55 0.30 0.99

# full model multivariable logistic regression analysis with DD yes/no as dependent variable, IRTmax dichotomised by the median, lame (locomotion score 3+4+5), and dried manure (cleanliness score 3+4) as independent variables, and farm as fixed effect

glm(formula = DDbin ~ factor(IRTmax_median) + factor(lame) + factor(dried manure) + factor(farm), family = "binomial", data = unwashed)

Deviance Residuals:

Min 1Q Median 3Q Max

-2.1375 -1.0753 0.6173 0.8378 1.6627

Coefficients:

Estimate Std. Error z value Pr(>|z|)

(Intercept) 1.2202 0.5011 2.435 0.01488 *

factor(IRTmax_median)1 0.9568 0.3712 2.578 0.00995 **

factor(lame)1 -0.8484 0.4866 -1.743 0.08128 .

factor(dried manure)1 0.2549 0.3410 0.747 0.45483

factor(farm)2 -0.8706 0.5729 -1.520 0.12860

factor(farm)3 -1.4652 0.5396 -2.715 0.00662 **

factor(farm)4 -0.6085 0.6107 -0.996 0.31905

---

Signif. codes: 0 ‘***’ 0.001 ‘**’ 0.01 ‘*’ 0.05 ‘.’ 0.1 ‘ ’ 1

(Dispersion parameter for binomial family taken to be 1)

Null deviance: 257.62 on 204 degrees of freedom

Residual deviance: 235.64 on 198 degrees of freedom

(324 observations deleted due to missingness)

AIC: 249.64

Number of Fisher Scoring iterations: 4

Single term deletions

Model:

DDbin ~ factor(IRTmax_median) + factor(lame) + factor(dried manure) + factor(farm)

Df Deviance AIC LRT Pr(>Chi)

<none> 235.64 249.64

factor(IRTmax_median) 1 242.52 254.52 6.8796 0.008719 **

factor(lame) 1 238.65 250.65 3.0111 0.082697 .

factor(dried manure) 1 236.21 248.21 0.5639 0.452708

factor(farm) 3 244.72 252.72 9.0771 0.028284 *

---

Signif. codes: 0 ‘***’ 0.001 ‘**’ 0.01 ‘*’ 0.05 ‘.’ 0.1 ‘ ’ 1

beta 2.5 % 97.5 %

(Intercept) 3.39 1.33 9.71

factor(IRTmax_median)1 2.60 1.27 5.48

factor(lame)1 0.43 0.16 1.12

factor(dried manure)1 1.29 0.67 2.55

factor(farm)2 0.42 0.13 1.24

factor(farm)3 0.23 0.08 0.64

factor(farm)4 0.54 0.16 1.79

# final reduced model multivariable logistic regression analysis with DD yes/no as dependent variable, IRTmax dichotomised by the median as independent variable, and farm as fixed effect

glm(formula = DDbin ~ factor(IRTmax_median) + factor(farm), family = "binomial", data = unwashed)

Deviance Residuals:

Min 1Q Median 3Q Max

-1.7245 -1.1374 0.7159 1.1032 1.2563

Coefficients:

Estimate Std. Error z value Pr(>|z|)

(Intercept) 0.4066 0.2784 1.461 0.1441

factor(IRTmax_median)1 0.3606 0.1919 1.879 0.0603 .

factor(farm)2 0.4633 0.3569 1.298 0.1942

factor(farm)3 -0.5014 0.3587 -1.398 0.1622

factor(farm)4 0.2302 0.4122 0.558 0.5766

factor(farm)5 -0.5902 0.2994 -1.971 0.0487 *

---

Signif. codes: 0 ‘***’ 0.001 ‘**’ 0.01 ‘*’ 0.05 ‘.’ 0.1 ‘ ’ 1

(Dispersion parameter for binomial family taken to be 1)

Null deviance: 717.62 on 528 degrees of freedom

Residual deviance: 686.18 on 523 degrees of freedom

AIC: 698.18

Number of Fisher Scoring iterations: 4

Single term deletions

Model:

DDbin ~ factor(IRTmax_median) + factor(farm)

Df Deviance AIC LRT Pr(>Chi)

<none> 686.18 698.18

factor(IRTmax_median) 1 689.71 699.71 3.5365 0.0600306 .

factor(farm) 4 707.81 711.81 21.6365 0.0002367 ***

---

Signif. codes: 0 ‘***’ 0.001 ‘**’ 0.01 ‘*’ 0.05 ‘.’ 0.1 ‘ ’ 1

beta 2.5 % 97.5 %

(Intercept) 1.50 0.88 2.63

factor(IRTmax_median)1 1.43 0.98 2.09

factor(farm)2 1.59 0.79 3.20

factor(farm)3 0.61 0.30 1.22

factor(farm)4 1.26 0.56 2.86

factor(farm)5 0.55 0.30 0.99

# WASHED FEET DATASET

# full model multivariable logistic regression analysis with DD yes/no as dependent variable, IRTmax, lame (locomotion score 3+4+5), and dried manure (cleanliness score 3+4) as independent variables, and farm as fixed effect

glm(formula = DDbin ~ IRTmax + factor(lame) + factor(dried manure) + factor(farm), family = "binomial", data = washed)

Deviance Residuals:

Min 1Q Median 3Q Max

-1.8290 -1.2151 0.7433 0.8519 1.4071

Coefficients:

Estimate Std. Error z value Pr(>|z|)

(Intercept) -1.49475 1.64617 -0.908 0.364

IRTmax 0.07218 0.05129 1.407 0.159

factor(lame)1 -0.35143 0.38556 -0.911 0.362

factor(dried manure)1 0.07677 0.30797 0.249 0.803

factor(farm)2 0.30133 0.41289 0.730 0.466

factor(farm)3 -0.40221 0.44377 -0.906 0.365

factor(farm)4 0.50787 0.49322 1.030 0.303

(Dispersion parameter for binomial family taken to be 1)

Null deviance: 289.64 on 228 degrees of freedom

Residual deviance: 277.51 on 222 degrees of freedom

(329 observations deleted due to missingness)

AIC: 291.51

Number of Fisher Scoring iterations: 4

Single term deletions

Model:

DDbin ~ IRTmax + factor(lame) + factor(dried manure) + factor(farm)

Df Deviance AIC LRT Pr(>Chi)

<none> 277.51 291.51

IRTmax 1 279.51 291.51 1.9983 0.1575

factor(lame) 1 278.33 290.33 0.8188 0.3655

factor(dried manure) 1 277.57 289.57 0.0623 0.8030

factor(farm) 3 282.25 290.25 4.7467 0.1913

beta 2.5 % 97.5 %

(Intercept) 0.22 0.01 5.61

IRTmax 1.07 0.97 1.19

factor(lame)1 0.70 0.33 1.52

factor(dried manure)1 1.08 0.59 1.99

factor(farm)2 1.35 0.60 3.04

factor(farm)3 0.67 0.28 1.59

factor(farm)4 1.66 0.64 4.51

# final reduced model multivariable logistic regression analysis with DD yes/no as dependent variable, IRTmax as independent variable, and farm as fixed effect

glm(formula = DDbin ~ IRTmax + factor(farm), family = "binomial", data = washed)

Deviance Residuals:

Min 1Q Median 3Q Max

-1.9435 -1.1860 0.6852 1.0679 1.6166

Coefficients:

Estimate Std. Error z value Pr(>|z|)

(Intercept) -3.65472 1.13222 -3.228 0.00125 **

IRTmax 0.12541 0.03477 3.607 0.00031 ***

factor(farm)2 0.97746 0.33236 2.941 0.00327 **

factor(farm)3 0.28091 0.35505 0.791 0.42884

factor(farm)4 0.88936 0.39457 2.254 0.02420 *

factor(farm)5 -0.22272 0.27175 -0.820 0.41246

---

Signif. codes: 0 ‘***’ 0.001 ‘**’ 0.01 ‘*’ 0.05 ‘.’ 0.1 ‘ ’ 1

(Dispersion parameter for binomial family taken to be 1)

Null deviance: 755.53 on 557 degrees of freedom

Residual deviance: 706.03 on 552 degrees of freedom

AIC: 718.03

Number of Fisher Scoring iterations: 4

Single term deletions

Model:

DDbin ~ IRTmax + factor(farm)

Df Deviance AIC LRT Pr(>Chi)

<none> 706.03 718.03

IRTmax 1 719.77 729.77 13.737 0.0002102 ***

factor(farm) 4 734.84 738.84 28.810 8.544e-06 ***

---

Signif. codes: 0 ‘***’ 0.001 ‘**’ 0.01 ‘*’ 0.05 ‘.’ 0.1 ‘ ’ 1

beta 2.5 % 97.5 %

(Intercept) 0.03 0.00 0.23

IRTmax 1.13 1.06 1.22

factor(farm)2 2.66 1.39 5.15

factor(farm)3 1.32 0.66 2.67

factor(farm)4 2.43 1.14 5.39

factor(farm)5 0.80 0.47 1.36

# full model multivariable logistic regression analysis with DD yes/no as dependent variable, IRTmax dichotomised by the median, lame (locomotion score 3+4+5), and dried manure (cleanliness score 3+4) as independent variables, and farm as fixed effect

glm(formula = DDbin ~ factor(IRTmax_median) + factor(lame) + factor(dried manure) + factor(farm), family = "binomial", data = washed)

Deviance Residuals:

Min 1Q Median 3Q Max

-1.8202 -1.2014 0.7327 0.8354 1.2993

Coefficients:

Estimate Std. Error z value Pr(>|z|)

(Intercept) 0.59989 0.40307 1.488 0.137

factor(IRTmax_median)1 0.27331 0.31329 0.872 0.383

factor(lame)1 -0.33818 0.38635 -0.875 0.381

factor(dried manure)1 0.09294 0.30722 0.303 0.762

factor(farm)2 0.30490 0.41249 0.739 0.460

factor(farm)3 -0.54368 0.42364 -1.283 0.199

factor(farm)4 0.47863 0.49324 0.970 0.332

(Dispersion parameter for binomial family taken to be 1)

Null deviance: 289.64 on 228 degrees of freedom

Residual deviance: 278.75 on 222 degrees of freedom

(329 observations deleted due to missingness)

AIC: 292.75

Number of Fisher Scoring iterations: 4

Single term deletions

Model:

DDbin ~ factor(IRTmax_median) + factor(lame) + factor(dried manure) + factor(farm)

Df Deviance AIC LRT Pr(>Chi)

<none> 278.75 292.75

factor(IRTmax_median) 1 279.51 291.51 0.7584 0.38383

factor(lame) 1 279.50 291.50 0.7557 0.38466

factor(dried manure) 1 278.84 290.84 0.0918 0.76196

factor(farm) 3 285.69 293.69 6.9455 0.07366 .

---

Signif. codes: 0 ‘***’ 0.001 ‘**’ 0.01 ‘*’ 0.05 ‘.’ 0.1 ‘ ’ 1

beta 2.5 % 97.5 %

(Intercept) 1.82 0.84 4.09

factor(IRTmax_median)1 1.31 0.71 2.43

factor(lame)1 0.71 0.34 1.54

factor(dried manure)1 1.10 0.60 2.02

factor(farm)2 1.36 0.60 3.05

factor(farm)3 0.58 0.25 1.32

factor(farm)4 1.61 0.62 4.38

# final reduced model multivariable logistic regression analysis with DD yes/no as dependent variable, IRTmax dichotomised by the median as independent variable, and farm as fixed effect

glm(formula = DDbin ~ factor(IRTmax_median) + factor(farm), family = "binomial", data = washed)

Deviance Residuals:

Min 1Q Median 3Q Max

-1.7811 -1.1907 0.6768 1.1309 1.2340

Coefficients:

Estimate Std. Error z value Pr(>|z|)

(Intercept) 0.2225 0.2676 0.831 0.40574

factor(IRTmax_median)1 0.1634 0.1861 0.878 0.37974

factor(farm)2 0.9713 0.3300 2.943 0.00325 **

factor(farm)3 -0.1121 0.3353 -0.334 0.73811

factor(farm)4 0.7533 0.3909 1.927 0.05396 .

factor(farm)5 -0.3546 0.2696 -1.315 0.18843

---

Signif. codes: 0 ‘***’ 0.001 ‘**’ 0.01 ‘*’ 0.05 ‘.’ 0.1 ‘ ’ 1

(Dispersion parameter for binomial family taken to be 1)

Null deviance: 755.53 on 557 degrees of freedom

Residual deviance: 718.99 on 552 degrees of freedom

AIC: 730.99

Number of Fisher Scoring iterations: 4Single term deletions

Model:

DDbin ~ factor(IRTmax_median) + factor(farm)

Df Deviance AIC LRT Pr(>Chi)

<none> 718.99 730.99

factor(IRTmax_median) 1 719.77 729.77 0.771 0.3799

factor(farm) 4 752.08 756.08 33.090 1.145e-06 ***

---

Signif. codes: 0 ‘***’ 0.001 ‘**’ 0.01 ‘*’ 0.05 ‘.’ 0.1 ‘ ’ 1

beta 2.5 % 97.5 %

(Intercept) 1.25 0.74 2.12

factor(IRTmax_median)1 1.18 0.82 1.70

factor(farm)2 2.64 1.39 5.09

factor(farm)3 0.89 0.46 1.73

factor(farm)4 2.12 1.00 4.67

factor(farm)5 0.70 0.41 1.19
