# Supplementary material for: Broad-spectrum infrared thermography for detection of M2 digital dermatitis lesions on hind feet of standing dairy cattle
Source: PLoS One. 2023 Jan 17;18(1):e0280098. doi: 10.1371/journal.pone.0280098 (PMC9844892; doi:10.1371/journal.pone.0280098)
Supplement: Attachment — Submitted filename: Comments.docx [file pone.0280098.s004.docx]

Dear Editor, Dr. Julio Souza

Thank you for your request dated 13 July 2022 concerning the evaluation of manuscript number PONE-D-22-12645, entitled (Broad-spectrum infrared thermography for detection of M2 digital dermatitis lesions on hind feet of standing dairy cattle). Bellow my comments on this manuscript:

**General comment**: Well written article, has viable information about topic of this manuscript.

**Title** of this manuscript is excellent. **The abstract** is covered this manuscript completely.

**Introduction**: readable, comprehensive, and covering the subject quite right. The first paragraph in the introduction should be based on a reference.

**Methodology**: perfect and good.

**Results**: good and clear. The titles of the tables in the results (Tables 1, 2, 3 and 4) are too long. I suggest that they be abbreviated.

**Discussion**: The results needs more discussion. The authors should mention the reasons for the results in detail.

**Conclusion**: good and clear.

**References**: excellent, clear and complete and modern and important references.

**Figure 1, 2 and 3** are not clear and need more accuracy.

Prof. Dr. Khalid Chillab Kridie Al-Salhie

Department of Animal production

College of Agriculture

University of Basrah.

Basrah city.

Iraq.

Tel:07723376572

07802671759

ORCID: <https://orcid.org/0000-0003-1121-7056>
